# Impact of Sulfurization Temperature on the Formation and Properties of Chalcogenide Perovskites

**DOI:** 10.3390/molecules30061198

**Published:** 2025-03-07

**Authors:** Pengnan Zhao, Lihuan Yang, Sen Kong, Haolei Hui, Lauren Samson, Kaiwei Guo, Bingyue Bian, Kaiyun Chen, Zhonghai Yu

**Affiliations:** 1 Key Laboratory of Magnetic Molecules and Magnetic Information Materials of Ministry of Education, School of Chemistry and Materials Science, Shanxi Normal University, Taiyuan 030006, China; 223111049@sxnu.edu.cn (P.Z.); 223111048@sxnu.edu.cn (L.Y.); ewe32e3@163.com (K.G.); bianbingyue@sxnu.edu.cn (B.B.); 2 School of Physics, Xi’an Jiaotong University, Xi’an 710049, China; mr.ks5767@stu.xjtu.edu.cn; 3 Department of Physics, University at Buffalo, The State University of New York, Buffalo, NY 14260, USA; haoleihu@buffalo.edu (H.H.); lsamson@buffalo.edu (L.S.); 4 Advanced Materials Research Central, Northwest Institute for Nonferrous Metal Research, Xi’an 710016, China; chenkaiyun309@gmail.com

**Keywords:** chalcogenide perovskite, temperature impact, photoluminescence

## Abstract

Chalcogenide perovskites have gained attention as alternative semiconductor materials, yet their experimental investigation remains limited. This study investigates the synthesis and characterization of a series of chalcogenide perovskite powder samples via the sulfurization of oxide precursors at different temperatures. Zr- and Hf-based chalcogenide perovskites adopted a perovskite structure with a *Pnma* space group, while Ti-based chalcogenides formed hexagonal phases. The minimum synthesis temperature varied among materials and was correlated with the strength of the A cation–oxygen bonds. The synthesized chalcogenide perovskites exhibit bandgaps suitable for solar cell absorption layers, and the photoluminescence (PL) results indicate that SrZrS_3_, SrHfS_3_, CaZrS_3_, and CaHfS_3_ are promising candidates for light-emitting semiconductors.

## 1. Introduction

Over the past two decades, significant progress has been made in photovoltaic and photoluminescent applications using organic–inorganic hybrid perovskite materials [1,2]. However, organic–inorganic halide perovskites face critical challenges, including susceptibility to water-induced decomposition, instability under light exposure [3], and the environmental and health risks posed by the inherent toxicity of lead-containing elements [4]. These issues urgently require solutions. To address these challenges, extensive efforts have been made, such as replacing organic groups at the A-site and lead at the B-site with alternatives like CsSnI_3_, which has shown some promising results [5]. However, the instability of divalent Sn makes it an unfavorable replacement option. To solve the problems mentioned above, some outstanding researchers have put forward several solutions. For example, the creative approach of encapsulating perovskite crystals in transparent glasses is a highly efficient way to address the issues of the poor stability and lead toxicity of perovskites in practical devices. This not only improves stability and reduces toxicity but also allows for the customization of optoelectronic properties, opening up new possibilities for perovskite-based optoelectronic and photonic applications [6]. Based on first-principles calculations, chalcogenide perovskites have been proposed as potential alternatives; it should be noted that while all group 16 elements of the periodic table are chalcogens, the term chalcogenide is more commonly used to refer to sulfides, selenides, and tellurides, rather than oxides [7]. Previous studies have explored various materials as precursors, and numerous advantages of this material system as a precursor have been presented [8,9,10]. Their bandgap and light absorption properties, calculated and verified experimentally, demonstrate potential for photovoltaic and light-emitting applications [11,12,13,14].

Currently, a wide range of synthesis techniques exists for chalcogenide perovskites, particularly sulfide perovskites, including the sulfurization of oxide perovskites, the sintering of binary sulfide powders, and solution-based methods [15,16,17,18]. Low-temperature synthesis is critical in advancing the practical applications of these materials [19]. Synthesizing high-quality BaZrS_3_ thin films from oxide targets using pulsed laser deposition (PLD) necessitates temperatures above 900 °C [20]. To enable low-temperature thin film deposition, using chalcogenide perovskite targets is a viable option, as shown in our previous studies [19,21]. To prepare these targets, sulfurizing oxide perovskite powder samples with CS_2_ remains a convenient method for synthesizing high-purity chalcogenide perovskites, as other solid-state reaction methods often introduce unwanted impurities and secondary phases. While several studies have investigated the synthesis and properties of specific chalcogenide perovskite powders at particular temperatures [22], a comprehensive comparison of the synthesis conditions, structures, and properties across different chalcogenide perovskite materials has yet to be conducted. In this study, we synthesized nine types of sulfide samples by sulfurizing oxide perovskites with CS_2_ at different temperatures to investigate the impact of temperature on the structure and properties of chalcogenide perovskites. Our results show that different materials require different minimum temperatures to form the chalcogenide perovskite structure with the *Pnma* space group. We categorized the products into three series: Ti-based, Zr-based, and Hf-based sulfides. While Zr-based and Hf-based compounds can form chalcogenide perovskites at specific temperatures, Ti-based compounds form hexagonal phases, which are consistent with earlier studies.

Our work expands on experimentally produced chalcogenide perovskite material systems exhibiting optoelectronic properties. In particular, CaZrS_3_ and CaHfS_3_ chalcogenide perovskite powders were reported for the first time. These materials were suggested to be promising thermoelectric materials based on first-principles calculations. Additionally, the optical properties of CaZr_1−x_Hf_x_S_3_ indicate its potential for photovoltaic applications [23,24].

## 2. Results and Discussion

In this work, we used CS_2_ to sulfurize oxide perovskites into chalcogenide perovskites with argon as the carrier gas. The sulfurization temperatures were adjusted for different oxide perovskites to obtain sulfide samples. The structure and stability of the perovskites were estimated using the Goldschmidt tolerance coefficient (***t***) and octahedral factor (***μ***) [25]. A stable distorted perovskite structure is expected when 0.71≤ ***t*** ≤0.91 and ***μ*** ≥ 0.41. Otherwise, non-perovskite structures, such as hexagonal or orthorhombic phases with a needle-like morphology, are likely to form. The previous literature shows that Zr- and Hf-based chalcogenide perovskites (ABS_3_) can form stable perovskite structures with a *Pnma* space group. However, the ***t*** for Ti-based chalcogenides is 0.33, which is below the threshold of 0.41 to form a stable perovskite structure [7,26]. Thus, attempts to fabricate Ti-based chalcogenide materials result in non-perovskite structures (see Figures S5 and S6). In the main text, we focus on the results of Zr- and Hf-based chalcogenide perovskites.

**Figure 1:**
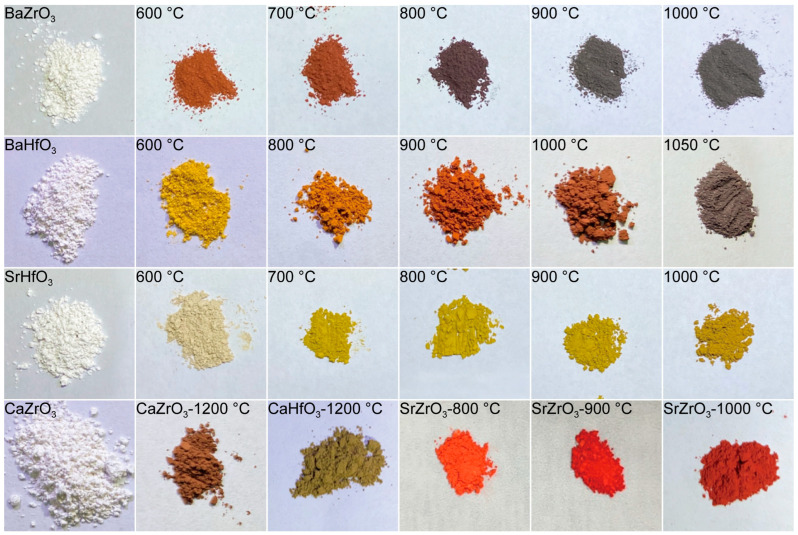
Photos of BaZrO_3_, BaHfO_3_, SrHfO_3_, CaZrO_3_, CaHfO_3_, and SrZrO_3_ powders, as well as these same powders (BaZrO_3_, BaHfO_3_, SrHfO_3_, CaZrO_3_, CaHfO_3_, and SrZrO_3_) sulfurized at different temperatures.

As shown in Figure 1, the sulfurized samples exhibit a clear trend of darkening color with increasing sulfurization temperature. Initially, all oxide perovskite powders are white, indicating minimal visible light absorption. However, as sulfurization temperature increases, the color of the powder samples progressively darkens. For example, the color transitions from white for BaZrO_3_ to light red when sulfurized at 600 °C, deepens to dark red at 700 °C, and turns to gray-black above 800 °C, suggesting strong absorption in the visible range. A similar darkening trend is observed in the other materials. BaHfO_3_ shifts from white to light yellow when sulfurized at 600 °C and becomes dark red at 1000 °C. SrHfO_3_, though showing a more gradual change, transitions from light yellow at 600 °C to dark yellow-green at 1000 °C. CaZrS_3_ sulfurized at 1200 °C appears dark red, while SrZrS_3_ sulfurized at 1000 °C shows a bright red hue. CaHfS_3_ samples processed at 1200 °C develop a dark yellow color, reinforcing the trend of increasing absorption with increasing sulfurization temperature. Even the non-perovskite-phase Ti-based samples sulfurized at 1000 °C exhibit a pronounced black appearance, as shown in Figure S5.

**Figure 2:**
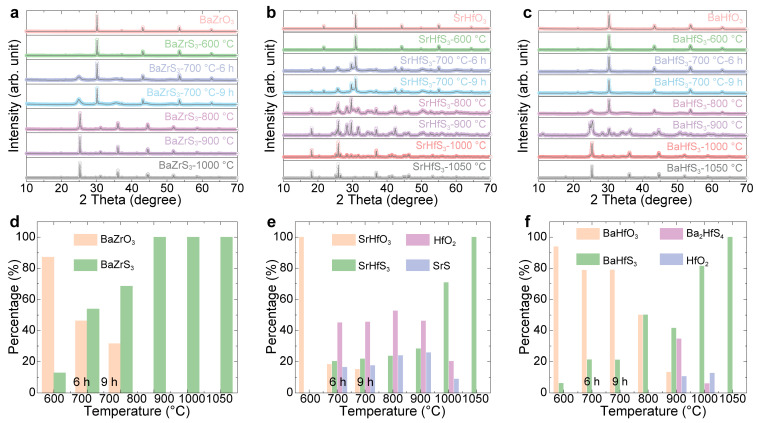
XRD patterns of (**a**) BaZrO_3_, (**b**) SrHfO_3_, and (**c**) BaHfO_3_ sulfurized at different temperatures and the molar percentage of the final products for (**d**) BaZrO_3_, (**e**) SrHfO_3_, and (**f**) BaHfO_3_ sulfurized at different temperatures.

To investigate the structural evolution of the products sulfurized at different temperatures, XRD was measured on these samples. The XRD patterns of BaZrO_3_, BaHfO_3_, and SrHfO_3_ sulfurized at various temperatures are shown in Figure 2a–c, respectively. The Rietveld refinement results for BaZrO_3_, BaHfO_3_, and SrHfO_3_ sulfurized at different temperatures are presented in Figures S1–S3, from which we extracted phase content and analyzed the relationship between phase composition and temperature. The proportions of oxide perovskites, chalcogenide perovskites, and intermediate phases at different temperature are shown in Figure 2d–f, respectively. From these results, the amount of oxide perovskite decreases with increasing sulfurization temperature for all samples, indicating the substitution of oxygen with sulfur. However, the minimum temperature required for complete sulfurization varies with the specific material. As seen in Figure 2d–f, BaZrS_3_ requires around 800 °C for a complete reaction, while SrHfS_3_ and BaHfS_3_ require temperatures exceeding 1000 °C. It is interesting to observe that BaZrO_3_ is converted into BaZrS_3_ directly with different fractions at different temperatures without any intermediate step. Earlier studies also suggest the presence of oxysulfides, as seen from the redshift in the absorption spectrum with increasing sulfurization temperature [15]. The oxysulfides are likely amorphous as they are not detected by XRD. In contrast, the sulfurization of SrHfO_3_ and BaHfO_3_ tended to form binary compounds at intermediate temperatures. (The refined XRD of SrZrS_3_, CaZrS_3_, and CaHfS_3_ can be found in Figure S4).

**Figure 3:**
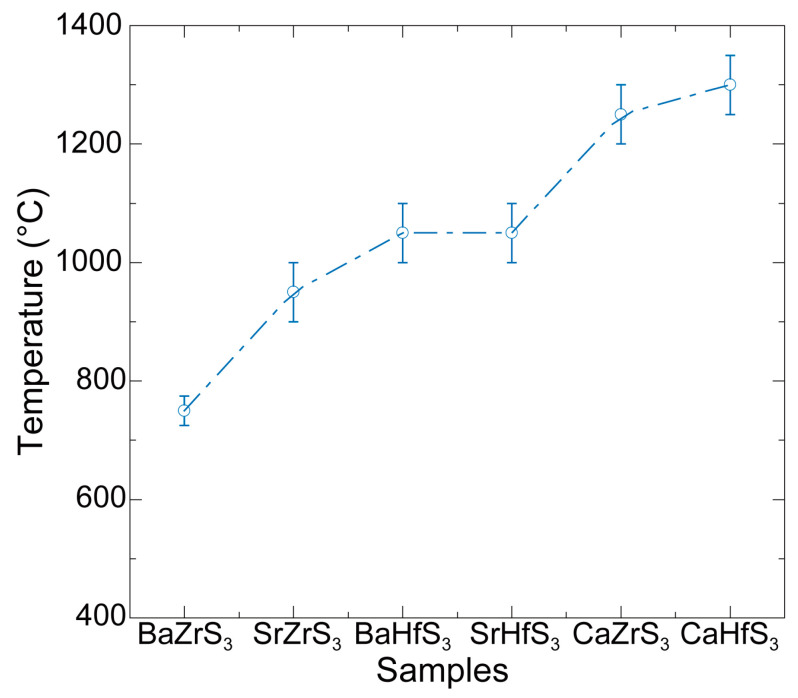
Minimum sulfurization temperatures for different oxide perovskites converted to chalcogenide perovskites.

For the Zr series, BaZrS_3_ requires 800 °C for complete sulfurization, SrZrS_3_ requires approximately 1000 °C, and CaZrS_3_ needs temperatures exceeding 1200 °C. Thus, there is a trend of requiring increased sulfurization temperature with decreasing size of the A cation. This trend also applies to the Hf series: both BaHfS_3_ and SrHfS_3_ require 1100 °C for complete sulfurization, while the yield of CaHfS_3_ reaches only about 74.15% even at 1200 °C. These results are summarized in Figure 3, which shows the approximate lowest sulfurization temperatures for the Zr- and Hf-based chalcogenide perovskites. The increasing sulfurization temperature with decreasing size of the A cation reflects the increasing strength of the A-O bonds in the oxide perovskite precursors as one moves down the alkaline earth metal group (Ba, Sr, Ca). This trend is directly related to the electronegativity of the alkaline earth metal. As electronegativity increases from Ba to Sr to Ca, the electronegativity difference between the A cation and O increases, strengthening the bonding (i.e., the bond becomes more ionic). Since the sulfurization of these oxides requires the breaking of the A-O bonds, more energy is required to break the Ca-O bond than the Ba-O bond, which is provided by the higher sulfurization temperature. Therefore, the formation of CaZrS_3_ requires the highest sulfurization temperature, while BaZrS_3_ requires the lowest.

**Figure 4:**
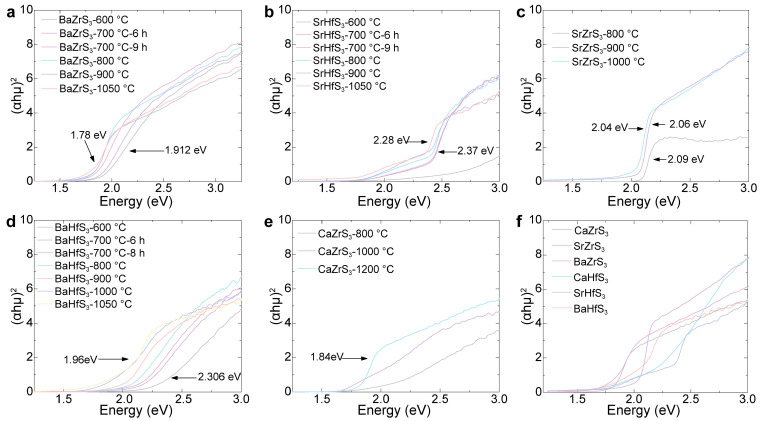
A Tauc plot of different samples sulfurized at different temperatures: (**a**) BaZrS_3_, (**b**) SrHfS_3_, (**c**) SrZrS_3_, (**d**) BaHfS_3_, and (**e**) CaZrS_3_. (**f**) A comparison of six sulfur-based chalcogenide perovskites’ bandgaps.

The bandgap of samples sulfurized at different temperatures was determined using UV-Vis spectroscopy. The variation in bandgap with sulfurization temperature is shown in Figure 4. For BaZrS_3_, SrHfS_3_, SrZrS_3_, and BaHfS_3_, the measured bandgap decreases as the sulfurization temperature increases, as illustrated in Figure 4a–d. This systematic redshift in bandgap, of the order of hundreds of meV, can be attributed to the presence of oxysulfide structural motifs in the partially sulfurized samples. If oxides and sulfides coexisted as separate phases, the UV-Vis measurements would reflect a superposition of two distinct band edge transitions instead of a smooth bandgap shift. The fact that XRD does not show an oxysulfide phase suggests that these oxysulfide structural motifs are not crystalline.

The measured bandgap values also correlate with the visual color of the samples at different temperatures. For samples sulfurized at high temperatures, the bandgap values for BaZrS_3_, SrZrS_3_, BaHfS_3_, and SrHfS_3_ match closely with theoretical calculations. However, the bandgap values for CaZrS_3_ and CaHfS_3_, shown in Figure 4e,f, are measured to be 1.84 eV and 2.13 eV, respectively, which are lower than the calculated values of 1.96 and 2.31 eV. This discrepancy is likely due to a high concentration of sulfur vacancies introduced at elevated temperatures, which alters the electronic structure and reduces the bandgap. Additional experiments are needed to determine the bandgap of CaZrS_3_ and CaHfS_3_ more accurately.

**Figure 5:**
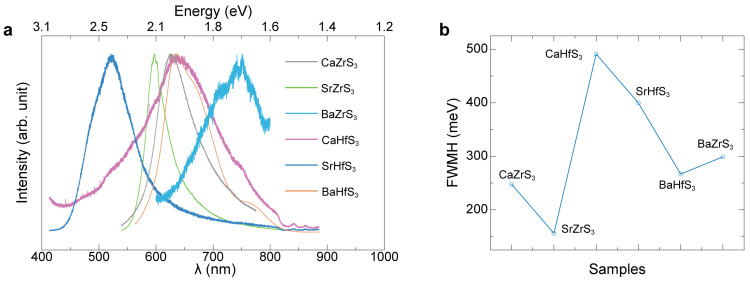
(**a**) The normalized PL spectra and (**b**) FWMH of the PL spectra of SrZrS_3_, CaZrS_3_, CaHfS_3_, SrHfS_3_, BaZrS_3_, and BaHfS_3_.

We further characterized the PL properties of the six Zr- and Hf-based perovskites. In Figure 5, we present normalized PL plots for easy comparison. Notably, all six materials exhibit PL in the visible range, with emission peaks ranging from 1.7 eV to 2.38 eV, confirming that they are all direct gap semiconductors. As we all know, emission peak values are closely related to bandgaps because they result from the radiative recombination of electrons and holes. When an electron in the conduction band recombines with a hole in the valence band, a photon is emitted, and its energy corresponds to the emission peak. However, defects and impurities in the materials are likely to have a certain impact on the bandgaps and emission characteristics. The emission peak values and bandgaps for the four Ba- and Sr-based perovskites are similar and closely align with theoretical predictions [7]. However, there are significant differences in the values of emission peaks and bandgaps for the two Ca-based perovskites, as shown in Table 1, which is derived from Figure 4f and Figure 5a. The full-width at half-maximum (FWMH) values can be seen in Figure 5b. SrZrS_3_ has the narrowest FWMH of 155.9 meV. This value is close to the 122 meV obtained from characterization [22], while CaHfS_3_ exhibits the highest FWHM of 491.1 meV. The measured PL peak of CaHfS_3_ is 1.93 eV. However, it has a relatively large FWHM. We speculate that this is because the final product is composed of 25.85% HfS_2_ and 74.15% CaHfS_3_. Bulk HfS_2_ exhibits an indirect bandgap, with reported values of 2.64 eV [27] or 2.58 eV [28]. As a result, the FWHM broadens towards the high-energy side, leading to its relatively large value.

## 3. Experimental Section

### 3.1. Synthesis of Chalcogenide Perovskite Powder Samples

We fabricated nine kinds of oxide perovskites for sulfurization following the ABO_3_ structure (A: Ca, Sr, and Ba; B: Ti, Zr, and Hf). All oxide perovskite ceramic samples were fabricated with a solid-state reaction method. First, ACO_3_ and BO_2_ were mixed with a stoichiometric ratio of 1:1 and ball milled for 8 h to ensure homogeneity. The mixture was then dried and placed into a muffle furnace for 8 h at 1200 °C. Next, the oxide perovskite ABO_3_ underwent sulfidation treatment at different temperatures in a tube furnace filled with carbon disulfide (CS_2_) and argon (Ar), following the procedure outlined in our previous work [29]. The different treatment conditions are as follows: sulfurization at 600 °C for 9 h, at 700 °C for 6 h, and at 800 °C for 4 h, or 2 h sulfidation at temperatures exceeding 900 °C.

### 3.2. Powder Characterization Techniques

The crystal diffraction patterns of powders were recorded by X-ray diffractometer (XRD, Bruker D8-Advance, Bruker AXS, Karlsruhe, Germany, Diffrac. Suite) equipped with a Cu Kα radiation source (λ =1.5418 Å). Measurements were performed under the θ–2θ scanning mode and continuous scanning with a step size of 0.02°. Raman spectra (Laser Raman Spectrometer, HORIBA, Tokyo, Japan, LabSpec6 version: 6.4.4.16) were obtained with a HORIBA Raman spectrometer using 532 nm laser excitation. The scanning electron microscopy (SEM) images and energy-dispersive X-ray spectrosco-py (EDX) analysis were acquired with a Focused Ion Beam-Scanning Electron Microscope (FIB-SEM, JSM-7000F, JEOL Japan Electronics Co., Ltd., Tokyo, Japan)-Carl Zeiss AURIGA CrossBeam with an Oxford EDS system. The absorption spectra were collected from a Cary series UV-Vis-NIR (Hitachi U-4100, Hitachi Limited, Tokyo, Japan) spectrophotometer measuring from 400 nm to 800 nm. Photoluminescence (PL, Laser Raman Spectrometer, HORIBA, Tokyo, Japan, LabSpec6 version: 6.4.4.16) was measured by a HORIBA Raman spectrometer with 325 nm and 532 nm lasers.

## 4. Conclusions

In conclusion, this study systematically investigated the impact of sulfurization temperature on the structural and optical properties of chalcogenide perovskites, among which CaHfS_3_ and CaZrS_3_ were reported for the first time. The minimum temperatures required for the formation of chalcogenide perovskites from different oxide precursors vary due to the different energy barriers that must be overcome during sulfur substitution. Breaking the Ca-O bond requires the highest energy, while Ba-O requires the lowest, and therefore CaZrS_3_ and CaHfS_3_ require the highest sulfurization temperatures. The six Zr- and Hf-based chalcogenide perovskite materials were found to have emission peaks ranging from 1.7 eV to 2.38 eV. The systematic studies of the series of chalcogenide perovskite materials offer valuable insights for their potential applications in optoelectronics.

## Figures and Tables

**Table 1:** Comparison of Zr- and Hf-based perovskite bandgap and PL peak.

Samples	Bandgap (eV)	PL Peak (eV)
BaZrS_3_	1.78	1.79
CaZrS_3_	1.84	1.98
BaHfS_3_	1.96	1.95
SrZrS_3_	2.04	2.08
CaHfS_3_	2.13	1.93
SrHfS_3_	2.28	2.28

## Data Availability

Data are contained within the article and Supplementary Materials.

## Supplementary Materials

The following supporting information can be downloaded at https://www.mdpi.com/article/10.3390/molecules30061198/s1: Figure S1: Plots of powder XRD patterns with Rietveld analysis. (a) BaZrO_3_ and BaZrO_3_ sulfurized at different temperatures: (b) 600 °C, (c) 700 °C for 6 h, (d) 700 °C for 9 h, (e) 800 °C, (f) 900 °C, and (g) 1000 °C; Figure S2: Plots of powder XRD patterns with Rietveld analysis. (a) SrHfO_3_ and SrHfO_3_ sulfurized at different temperatures: (b) 600 °C, (c) 700 °C for 6 h, (d) 700 °C for 9 h, (e) 800 °C, (f) 900 °C, (g) 1000 °C, and (h) 1050 °C; Figure S3: Plots of powder XRD patterns with Rietveld analysis. (a) BaHfO_3_ and BaHfO_3_ sulfurized at different temperatures: (b) 600 °C, (c) 700 °C for 6 h, (d) 700 °C for 9 h, (e) 800 °C, (f) 900 °C, (g) 1000 °C, and (h) 1050 °C; Figure S4: Plots of powder XRD patterns with Rietveld analysis. (a) SrZrO_3_ sulfurized at different temperatures: (b) 800 °C, (c) 900 °C, and (d) 1000 °C. (e) CaZrO_3_ and (f) CaZrO_3_ sulfurized at 1200 °C. (g) CaHfO_3_ and (h) CaHfO_3_ sulfurized at 1200 °C; Figure S5: Photos of CaTiO_3_, SrTiO_3_, and BaTiO_3_ powder as well as CaTiO_3_, SrTiO_3_, and BaTiO_3_ powder sulfurized at 1000 °C; Figure S6: Plots of powder XRD patterns with Rietveld analysis. (a) SrTiO_3_, (b) BaTiO_3_, (c) SrTiO_3_, and (d) BaTiO_3_ sulfurized at 1000 °C and (e) CaTiO_3_ and CaTiO_3_ sulfurized at 1000 °C; Figure S7: The room-temperature Raman spectra of (a) BaZrO_3_, (b) BaHfO_3_, and (c) SrHfO_3_ sulfurized at different temperatures. (d) The room-temperature Raman spectra of CaZrS_3_ sulfurized at 1200 °C, SrZrS_3_ sulfurized at 1000 °C, and CaHfS_3_ sulfurized at 1200 °C; Figure S8: The room-temperature Raman spectra of (a) SrTiO_3_ and (b) BaTiO_3_ sulfurized at 1000 °C; Figure S9: Typical SEM images of BaHfO_3_, BaZrO_3_, and CaZrO_3_ after sulfurization at different temperatures are presented as follows: For BaHfO_3_, images (a–c) correspond to sulfurization temperatures of 600 °C, 800 °C, and 1200 °C, respectively. For BaZrO_3_, images (d–f) correspond to sulfurization temperatures of 600 °C, 800 °C, and 1000 °C. And for CaZrO_3_, images (g–i) show the samples sulfurized at 800 °C, 1000 °C, and 1200 °C; Figure S10: The FWHMs of the (121) peak as a function of temperature. (a) BaZrO_3_, (b) SrHfO_3_, and (c) BaHfO_3_ sulfurized at different temperatures; Figure S11: (a) The ratios of S to S + O concentrations in various samples at different temperatures, along with the EDX spectra of (b) BaZrS_3_, (c) BaHfS_3_, (d) SrHfS_3_, and (e) CaZrS_3_ sulfurized at high temperatures.
