# Supplementary material for: Impact of Sulfurization Temperature on the Formation and Properties of Chalcogenide Perovskites
Source: Molecules. 2025 Mar 7;30(6):1198. doi: 10.3390/molecules30061198 (PMC11944344; doi:10.3390/molecules30061198)
Supplement: Supplementary file 1 [file molecules-30-01198-s001.zip › molecules-3519238-supplementary.pdf]

Supporting Information

Impact of Sulfurization Temperature on the Formation and Properties of Chalcogenide Perovskites

Pengnan Zhao [1,†], Lihuan Yang [1,†], Sen Kong [2], Haolei Hui [3], Lauren Samson [3], Kaiwei Guo [1], Bingyue Bian [1], Kaiyun Chen [4] and Zhonghai Yu [1,*]

[1] Key Laboratory of Magnetic Molecules and Magnetic Information Materials of Ministry of Education, School of Chemistry and Materials Science, Shanxi Normal University, Taiyuan 030006, China

[2] School of Physics, Xi'an Jiaotong University, Xi'an 710049, China

[3] Department of Physics, University at Buffalo, The State University of New York, Buffalo, NY 14260, USA

[4] Advanced Materials Research Central, Northwest Institute for Nonferrous Metal Research, Xi'an 710016, China

* Correspondence: yuzh@sxnu.edu.cn

† These authors contributed equally to this work.

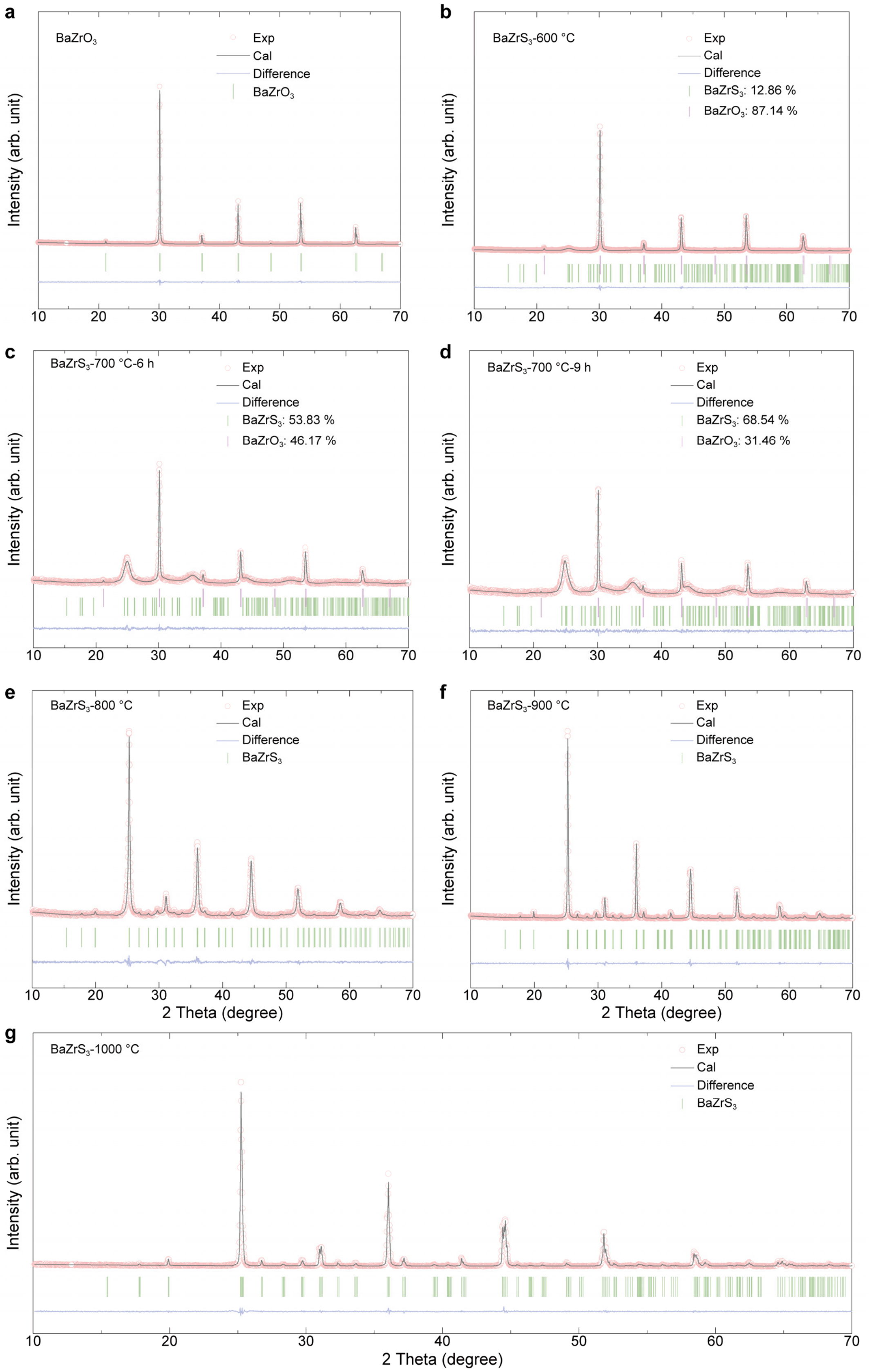

Figure S1. Plots of powder XRD patterns with Rietveld analysis. (**a**) $BaZrO_3$ and $BaZrO_3$ sulfurized at different temperatures: (**b**) 600 °C, (**c**) 700 °C for 6 h, (**d**) 700 °C for 9 h, (**e**) 800 °C, (**f**) 900 °C and (**g**) 1000 °C.

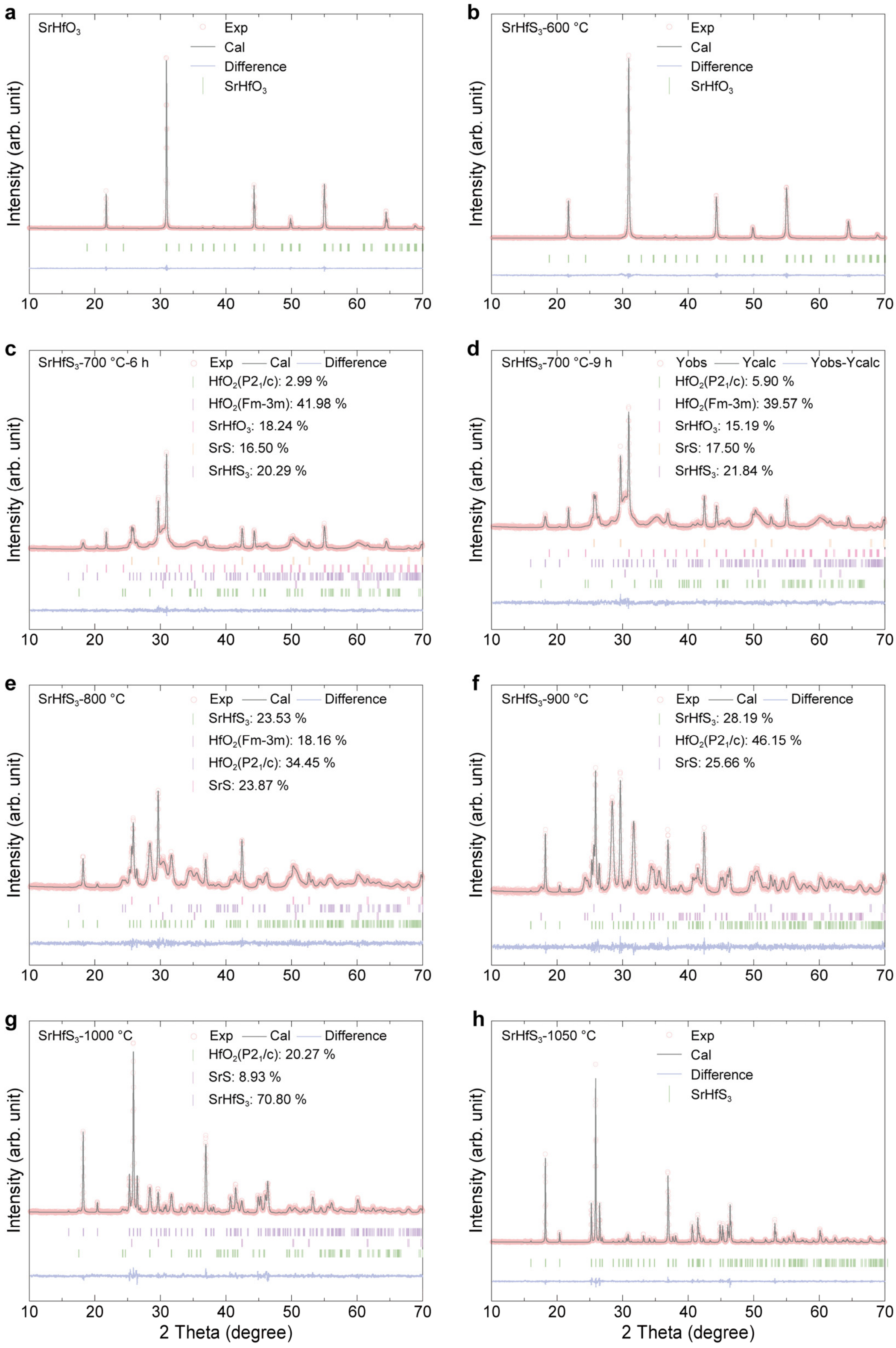

Figure S2. Plots of powder XRD patterns with Rietveld analysis. (**a**) $SrHfO_3$ and $SrHfO_3$ sulfurized at different temperatures: (**b**) 600 °C, (**c**) 700 °C for 6 h, (**d**) 700 °C for 9 h, (**e**) 800 °C, (**f**) 900 °C, (**g**) 1000 °C and (**h**) 1050 °C.

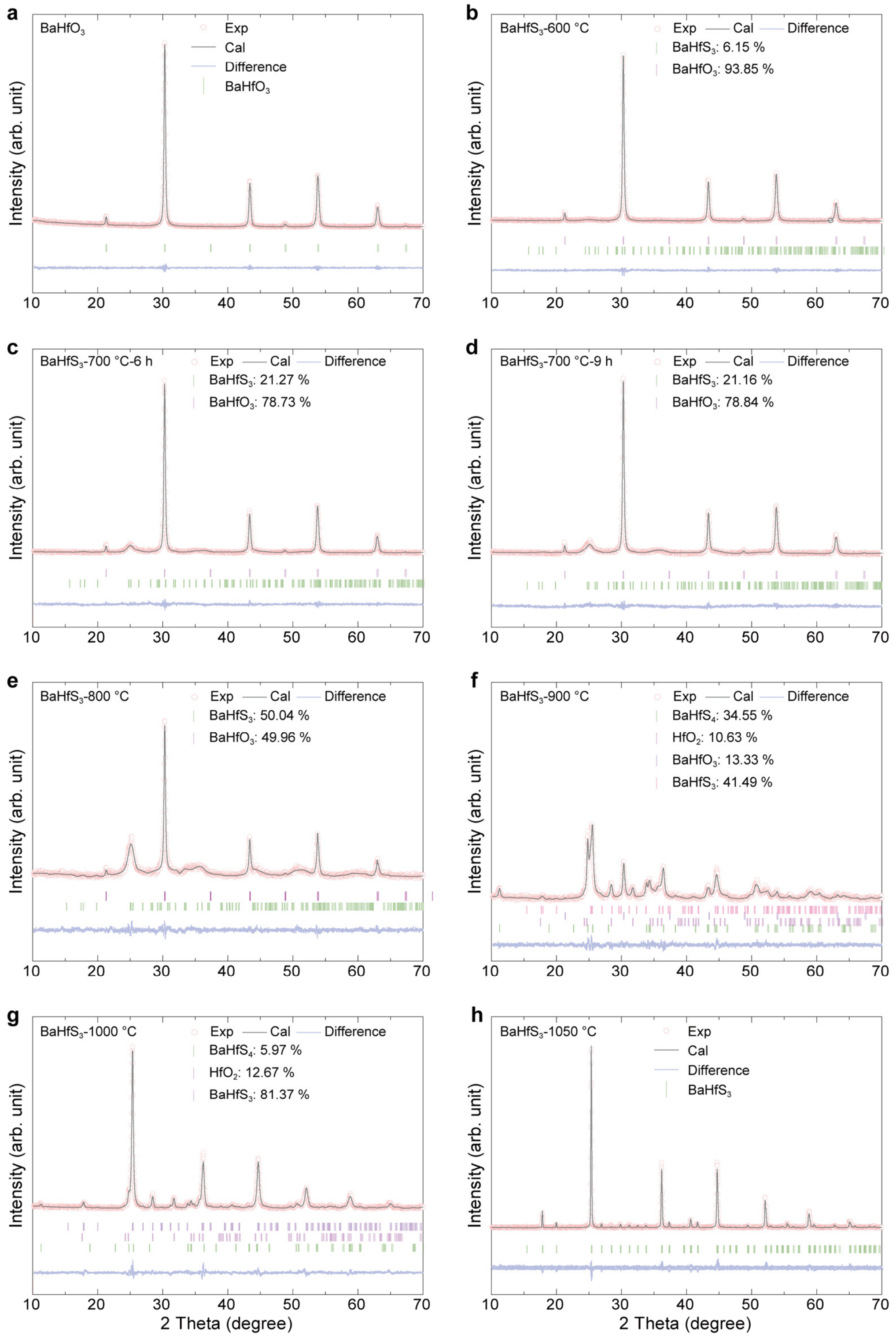

Figure S3. Plots of powder XRD patterns with Rietveld analysis. (**a**) $BaHfO_3$ and $BaHfO_3$ sulfurized at different temperatures: (**b**) 600 °C, (**c**) 700 °C for 6 h, (**d**) 700 °C for 9 h, (**e**) 800 °C, (**f**) 900 °C, (**g**) 1000 °C and (**h**) 1050 °C.

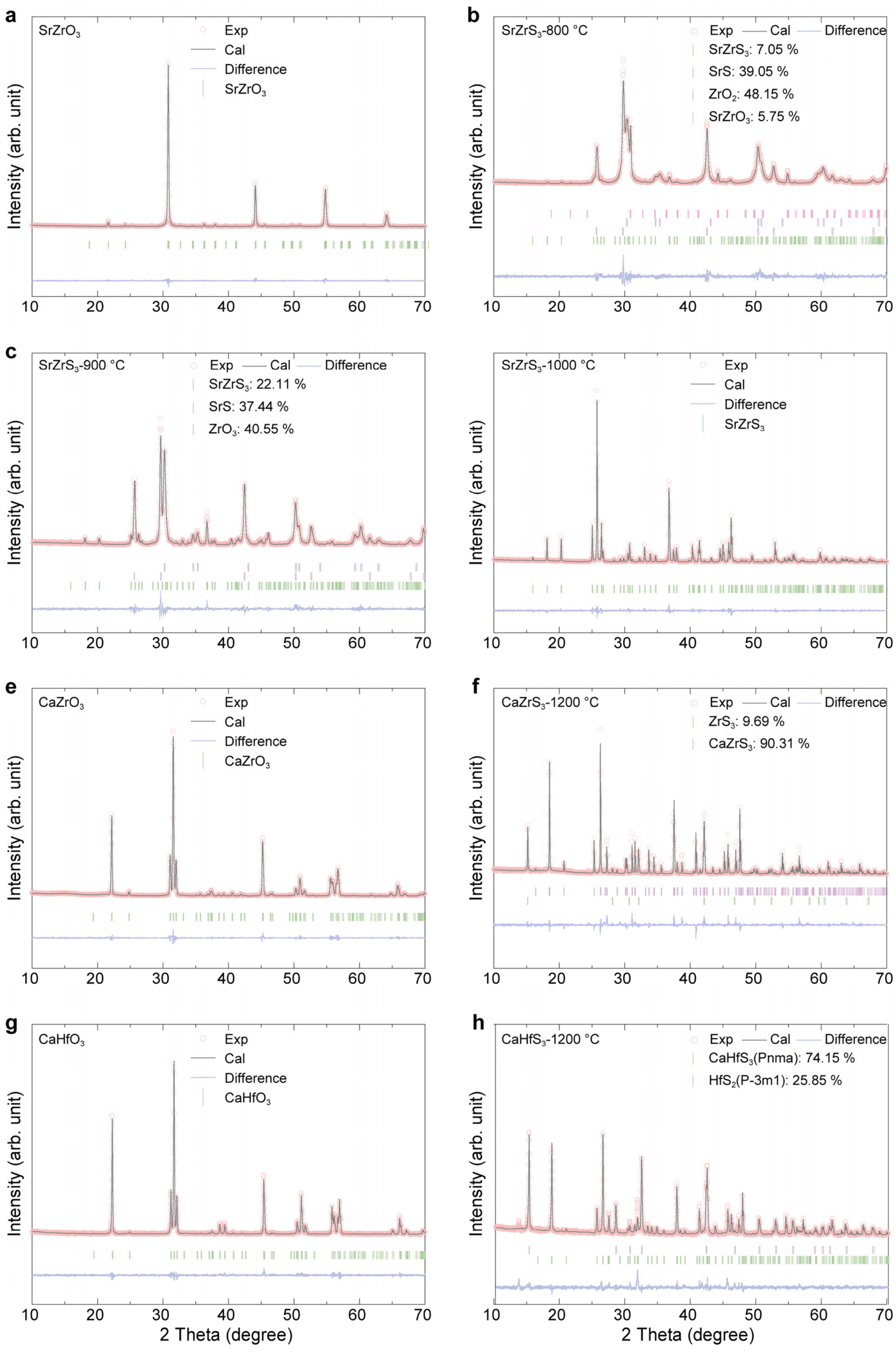

Figure S4. Plots of powder XRD patterns with Rietveld analysis. (**a**) $SrZrO_3$ sulfurized at different temperatures: (**b**) 800 °C, (**c**) 900 °C, (**d**) 1000 °C. (**e**) $CaZrO_3$, and (**f**) $CaZrO_3$ sulfurized at 1200 °C. (**g**) $CaHfO_3$ and (**h**) $CaHfO_3$ sulfurized at 1200 °C.

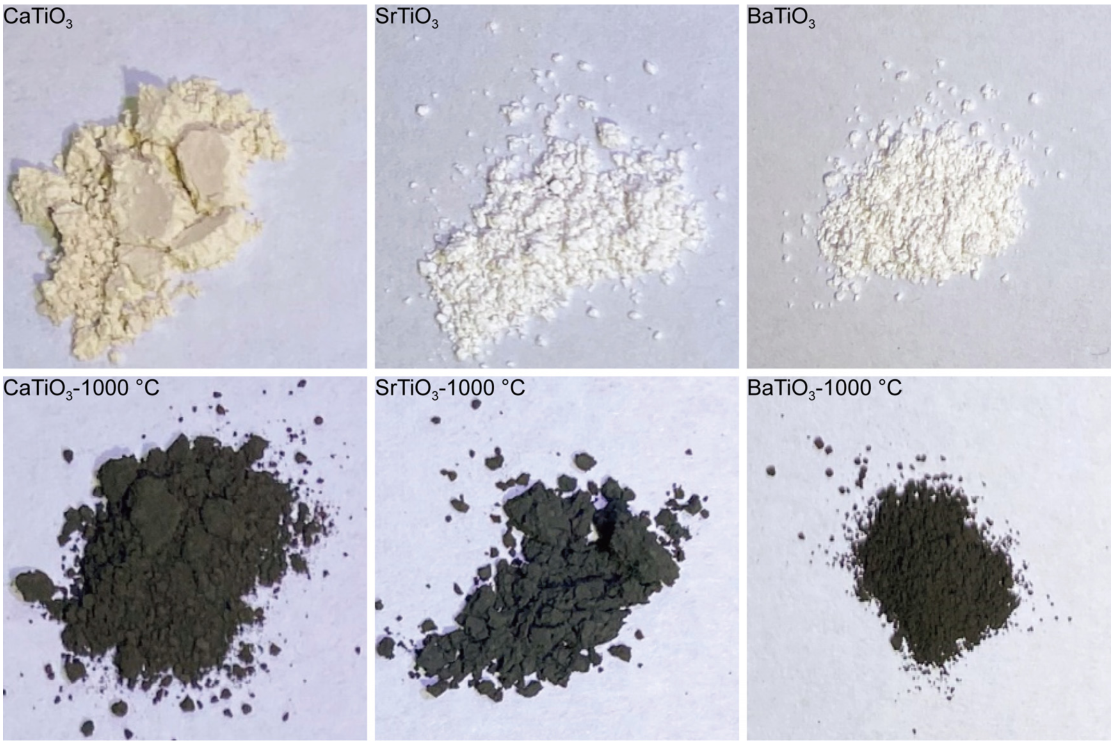

Figure S5. Photos of $CaTiO_3$, $SrTiO_3$ and $BaTiO_3$ powder as well as $CaTiO_3$, $SrTiO_3$ and $BaTiO_3$ powder sulfurized at 1000 °C.

Regarding the XRD of Ti-based chalcogenide perovskite, as shown in Figure S6, we can see that the XRD patterns of $BaTiS_3$ and $SrTiS_3$ sulfured at 1000 °C do not correspond to the perovskite structure but to the hexagonal phase, the structure of $CaTiS_3$ cannot be confirmed because there is not experimental and calculated data.

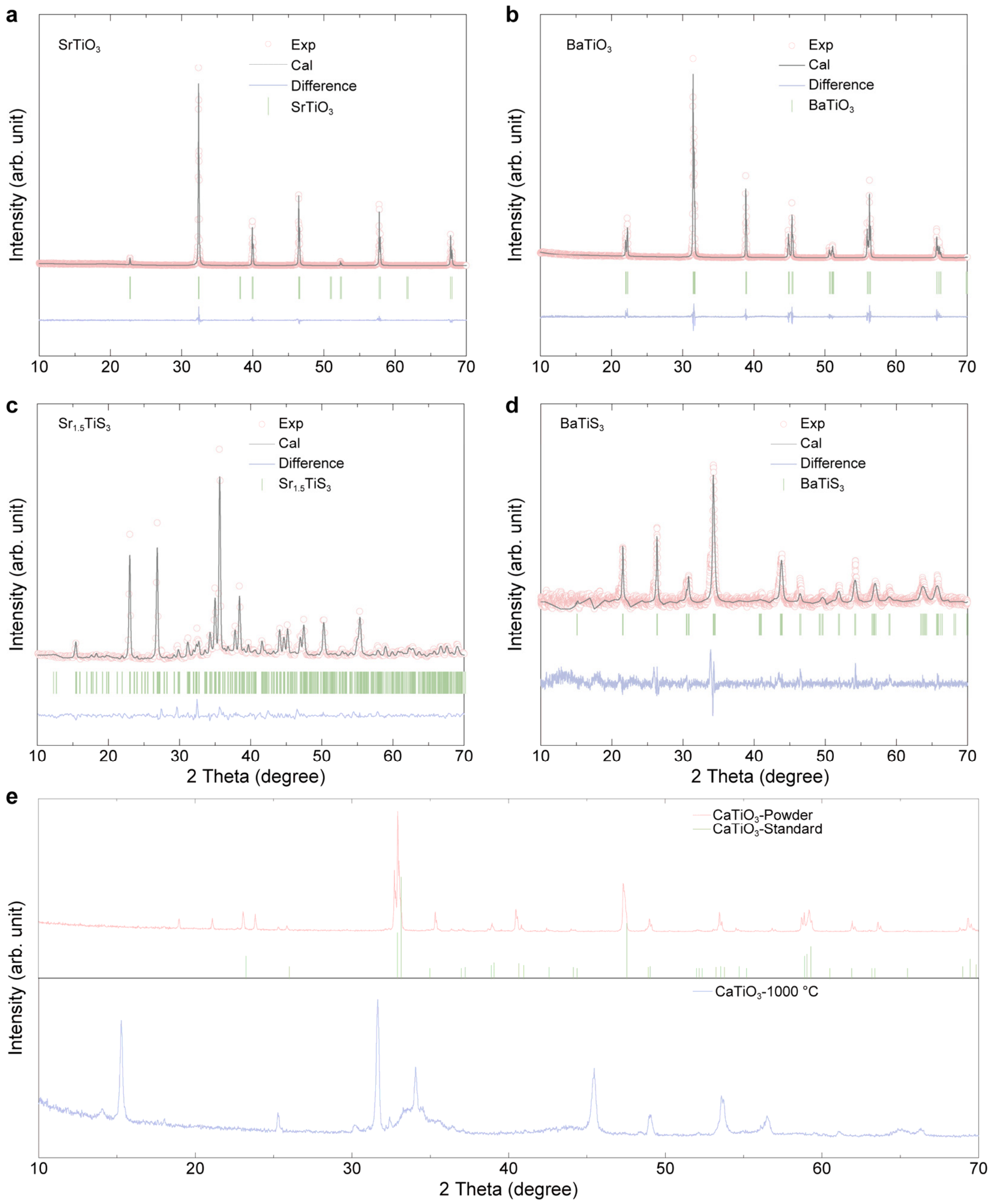

Figure S6. Plots of powder XRD patterns with Rietveld analysis. (**a**) $SrTiO_3$, (**b**) $BaTiO_3$, (**c**) $SrTiO_3$ and (**d**) $BaTiO_3$ sulfurized at 1000 °C, (**e**) $CaTiO_3$ and $CaTiO_3$ sulfurized at 1000 °C.

To confirm the structure of the materials we sulfurized at different temperatures, we also measured the Raman spectra of these six kinds of chalcogenide perovskites at room temperature. The Raman spectra of $BaZrS_3$ and $BaHfS_3$ samples sulfured at different temperatures are shown in Figure S7. Although the peaks are not sharp, the identifiable peaks correspond to the perovskite structure with the *Pnma* space group. For $SrHfS_3$, it is obvious that the peaks of samples sulfured from 700 °C–1050 °C match with those previously reported $SrHfS_3$. Although the $SrHfS_3$ is not the dominant phase in the product. The Raman spectra of the $SrZrS_3$ also matches with the work that has been published before. Regarding the Raman spectra of $CaZrS_3$ and $CaHfS_3$, as this is the first time they have been measured, the spectra are similar to those of $SrZrS_3$ and $SrHfS_3$. We inferred that these peaks belonged to the perovskite structure of the $CaZrS_3$ and $CaHfS_3$ which is beneficial for further research on these materials. The Raman spectra of the $BaTiS_3$ and $SrTiS_3$ sulfurized at 1000 °C are shown in Figure S8.

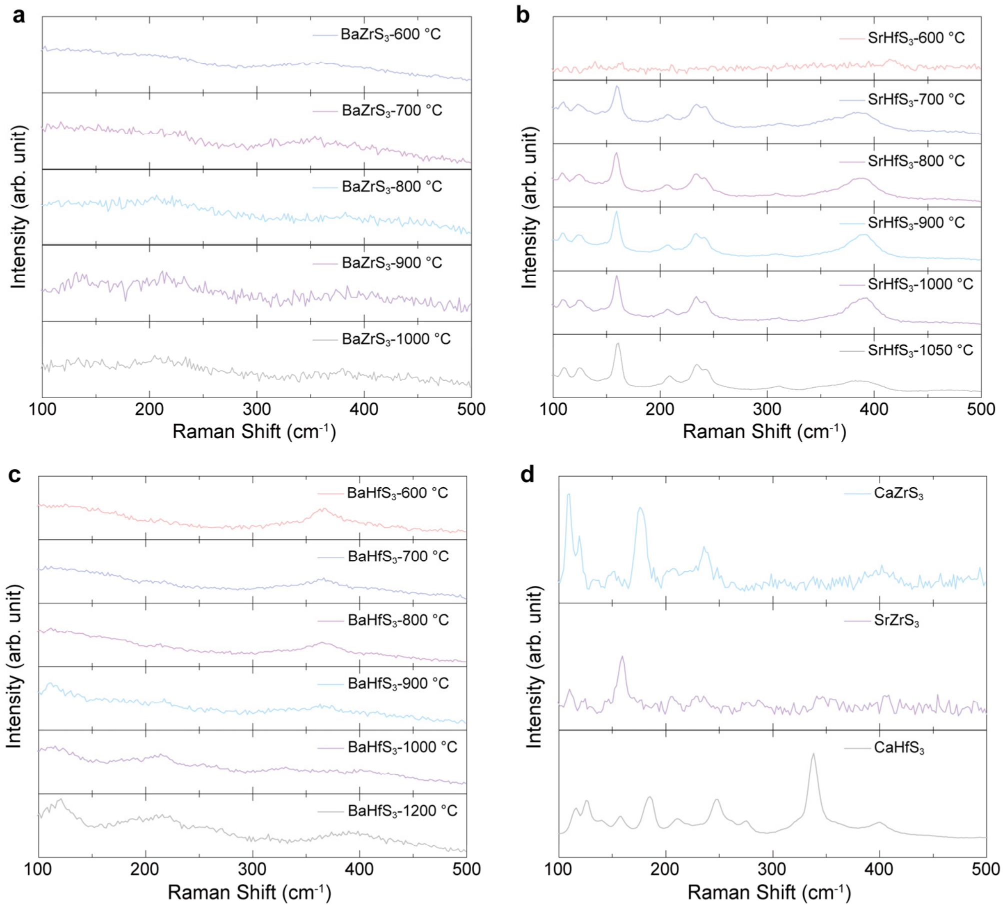

Figure S7. The room temperature Raman spectra of (**a**) $BaZrO_3$, (**b**) $BaHfO_3$ and (**c**) $SrHfO_3$ sulfurized at different temperatures. (**d**) The room temperature Raman spectra of $CaZrS_3$ sulfurized at 1200 °C, $SrZrS_3$ sulfurized at 1000 °C and $CaHfS_3$ sulfurized at 1200 °C.

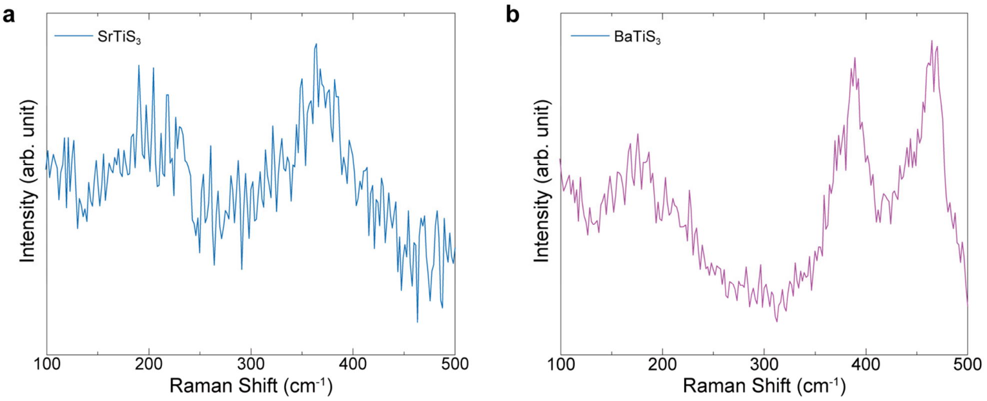

Figure S8. The room temperature Raman spectra of (**a**) $SrTiO_3$ and (**b**) $BaTiO_3$ sulfurized at 1000 °C.

SEM images of four types of chalcogenide perovskites are presented in Figure S9. The crystal size of the chalcogenide perovskites increases with increasing temperature which is consistent with the result calculated by XRD in Figure S10a–c. The ratio of S/(S+O) concentrations can be seen in Figure S11, we can see that the ratio of S/(S+O) concentrations increases from about 15% to 100% with increasing temperature this is the procedure of replacement of oxygen atoms by sulfur at high temperatures. Regarding the $CaZrS_3$, although

the oxygen is completely substituted with sulfur, the final product is not only $CaZrS_3$ but also consists of binary ZrS_2. The EDX spectra of these chalcogenide perovskites can be seen in Figure S11. From Figure S11b–e, it can be observed that for each sample, the relative proportion of the three elements can be determined. Through the corresponding element ratio in Figure S11, it is known that the proportion of cations is close to 1:1. However, in the final synthesized chalcogenide perovskite compound, the ratio of sulfur to its corresponding cations is not 1:1:3 because there is a significant sulfur deficiency, leading to the presence of some sulfur vacancies. These sulfur vacancies are consistent with previous research findings and indicate that the occurrence of sulfur vacancies may be inevitable during the synthesis of chalcogenide perovskite compounds under atmospheric pressure and high-temperature conditions.

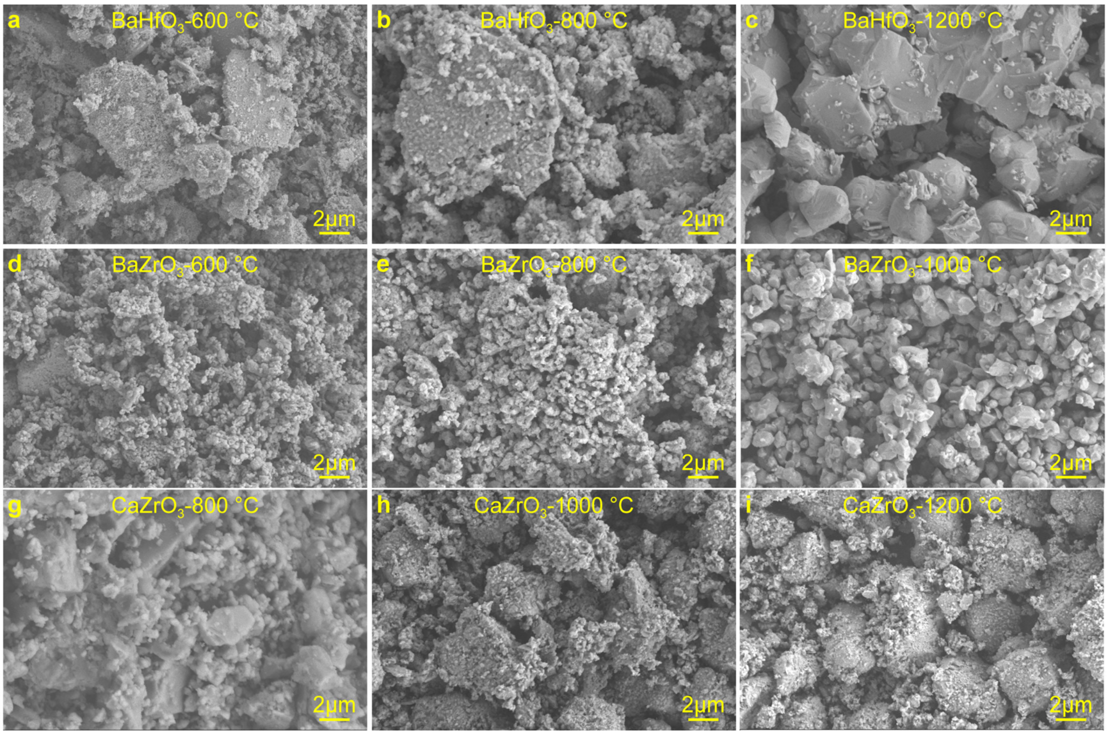

Figure S9. Typical SEM images of $BaHfO_3$, $BaZrO_3$, and $CaZrO_3$ after sulfurization at different temperatures are presented as follows: For $BaHfO_3$, images (**a**–**c**) correspond to sulfurization temperatures of 600 °C, 800 °C, and 1200 °C respectively. For $BaZrO_3$, images (**d**–**f**) correspond to sulfurization temperatures of 600 °C, 800 °C, and 1000 °C. And for $CaZrO_3$, images (**g**–**i**) show the samples sulfurized at 800 °C, 1000 °C, and 1200 °C.

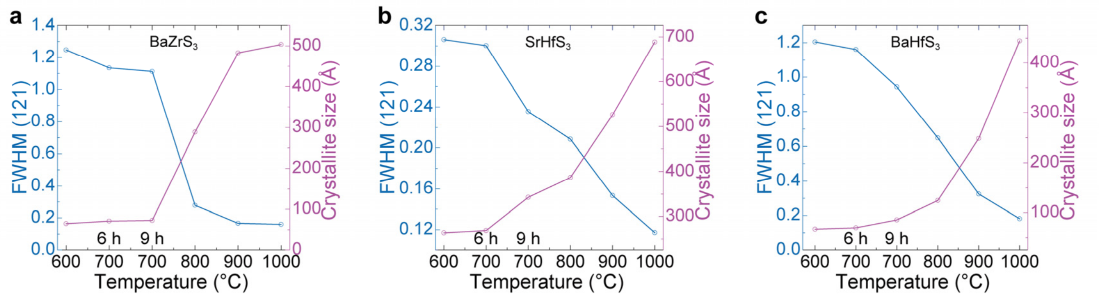

Figure S10. The FWHMs of the (121) peak as a function of temperature. (**a**) $BaZrO_3$, (**b**) $SrHfO_3$ and (**c**) $BaHfO_3$ sulfurized at different temperatures.

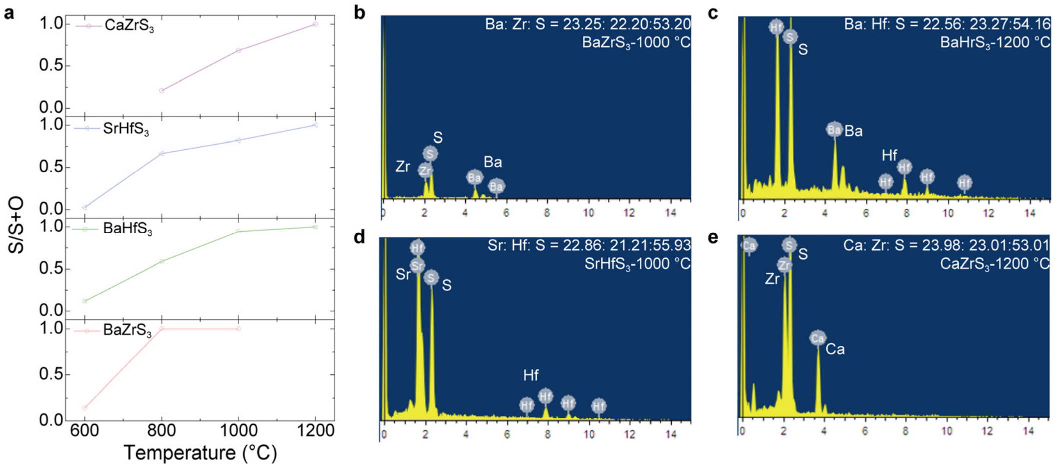

Figure S11. (**a**) The ratios of S to S + O concentrations in various samples at different temperatures, along with the EDX spectra of (**b**) $BaZrS_3$, (**c**) $BaHfS_3$, (**d**) $SrHfS_3$, and (**e**) $CaZrS_3$ sulfurized at high temperatures.
